# Effects of multi-domain cognitive training on working memory retrieval in older adults: behavioral and ERP evidence from a Chinese community study

**DOI:** 10.1038/s41598-020-79784-z

**Published:** 2021-01-13

**Authors:** Xiangfei Hong, You Chen, Jijun Wang, Yuan Shen, Qingwei Li, Binglei Zhao, Xiaoli Guo, Wei Feng, Wenyuan Wu, Chunbo Li

**Affiliations:** 1 Shanghai Key Laboratory of Psychotic Disorders, Shanghai Mental Health Center, Shanghai Jiao Tong University School of Medicine, 600 Wan Ping Nan Road, Shanghai, 200030 People’s Republic of China; 2 Shanghai Yangpu District Mental Health Center, Shanghai, 200090 People’s Republic of China; 3 CAS Center for Excellence in Brain Science and Intelligence Technology (CEBSIT), Chinese Academy of Science, Shanghai, People’s Republic of China; 4 Brain Science and Technology Research Center, Shanghai Jiao Tong University, Shanghai, 200030 People’s Republic of China; 5 Institute of Psychology and Behavioral Science, Shanghai Jiao Tong University, Shanghai, 200030 People’s Republic of China; 6 Department of Psychiatry, Shanghai Tenth People’s Hospital, Tongji University School of Medicine, Shanghai, 200072 People’s Republic of China; 7 Department of Psychiatry, Tongji Hospital, Tongji University, Shanghai, 200065 People’s Republic of China; 8 School of Biomedical Engineering, Shanghai Jiao Tong University, Shanghai, People’s Republic of China; 9 Department of Psychological Medicine, Renji Hospital, Shanghai Jiao Tong University School of Medicine, 160 Pujian Road, Shanghai, 200127 People’s Republic of China

**Keywords:** Psychology, Cognitive ageing, Cognitive neuroscience

## Abstract

Working memory (WM) is a fundamental cognitive function that typically declines with age. Previous studies have shown that targeted WM training has the potential to improve WM performance in older adults. In the present study, we investigated whether a multi-domain cognitive training program that was not designed to specifically target WM could improve the behavioral performance and affect the neural activity during WM retrieval in healthy older adults. We assigned healthy older participants (70–78 years old) from a local community into a training group who completed a 3-month multi-domain cognitive training and a control group who only attended health education lectures during the same period. Behavioral and electroencephalography (EEG) data were recorded from participants while performing an untrained delayed match or non-match to category task and a control task at a pre-training baseline session and a post-training follow-up session. Behaviorally, we found that participants in the training group showed a trend toward greater WM performance gains than participants in the control group. Event-related potential (ERP) results suggest that the task-related modulation of P3 during WM retrieval was significantly enhanced at the follow-up session compared with the baseline session, and importantly, this enhancement of P3 modulation was only significant in the training group. Furthermore, no training-related effects were observed for the P2 or N2 component during WM retrieval. These results suggest that the multi-domain cognitive training program that was not designed to specifically target WM is a promising approach to improve WM performance in older adults, and that training-related gains in performance are likely mediated by an enhanced modulation of P3 which might reflect the process of WM updating.

## Introduction

It is well-known that many high level cognitive functions significantly decline with age, including working memory (WM), attention, long-term memory, task switching and processing speed^1,2^. Developing targeted and effective intervention approaches to aid cognition in older adults has emerged as a center research topic in cognitive neuroscience^3–5^. As a fundamental cognitive ability, WM stores and manipulates information for ongoing cognition during short periods of time, which is required for a wide range of cognitive functions and real-world activities^6^. Based on this perspective, WM has captured great attention in cognitive training research, and many training programs have been designed to specifically target WM^7^. For example, previous studies have shown that training older adults in certain WM tasks during a relatively short period (e.g., 10 days, 5 weeks or 3 months) could lead to a significant improvement in behavioral performance (i.e., accuracy) during the trained or untrained WM tasks, something referred to as ‘near’ transfer effects^8–15^. On the other hand, ‘far’ transfer is typically used to describe situations in which the training and transfer tasks are not regarded to rely on the same core process, while the distinction between what is ‘far’ and what is ‘near’ is usually qualitative rather than quantitative^16,17^. Considering the fundamental role of WM in human fluid cognition, a natural assumption would be that training WM should also produce far transfer effects, which has been demonstrated by previous research, although the effects might not be as conclusive as that in near transfer^14,18–20^. In contrast, few studies have examined whether cognitive training programs that are not designed to specifically target WM can improve WM ability, presumably because WM is believed to be a central mental capacity and is usually prioritized as the primary training purpose^21^.

Although cognitive training has been shown to be effective in improving the specifically targeted cognitive functions (i.e., WM), such single-domain training program largely neglects the complicated interactions between multiple cognitive processes required for a viable and healthy mental state^22^. Therefore, in our previous work, we designed a multi-domain cognitive training program that covered a variety of cognitive domains (i.e., memory, reasoning and problem-solving strategies) and physical exercise^23^. Through a randomized controlled trial in which participants were randomly assigned to three groups, i.e., a multi-domain training group, a single-domain training group, and a wait list control group, we found that participants who received multi-domain cognitive training showed improvements on the Repeatable Battery for the Assessment of Neuropsychological Status (RBANS) total score, the index scores of visual reasoning, and immediate and delayed memory^23^. Compared with single-domain training approach that only covered reasoning, multi-domain training leaded to more benefits to the memory ability^23^. Furthermore, using magnetic resonance imaging (MRI), we have shown the training-related effects on resting state brain activity^24–26^ and cortical thickness^27^ in healthy older adults.

However, given that the memory-related training in our multi-domain training program was adopted from neuropsychological tests (i.e., the word learning test and digit span test) instead of WM tasks (i.e., N-back or delayed match to sample task) that were usually applied in previous studies^7,28^, it still remains unclear whether the training effects could transfer to WM, a central component of human fluid cognition. Thus, the first goal of the present study was to directly test whether this 3-month multi-domain cognitive training could improve the WM performance in healthy older adults. To this end, we had healthy older adults assigned into a training group and a control group, and recorded their behavioral performance during an untrained delayed match or non-match to category (DMC/DNMC) task at a pre-training and a post-training session. Based on our previous study^23^, we expected to observe significant training-related improvements in WM performance. Moreover, the training-related performance gains might be different between the DMC and the DNMC task. Specifically, although both tasks require participants to hold a visual stimulus in WM over a delay period before making a choice response, participants should select the familiar stimulus in DMC, while select the novel stimulus in DNMC. In adult humans, unlike children and animals, matching is a more natural process than non-matching, and the instinctive response at the choice stage is to the familiar rather than the novel^29–31^. In other words, making the choice in DNMC involves inhibiting an instinctively response tendency in order to make a correct choice, and thus DNMC is harder than DMC for adult humans^29^. Therefore, we would expect that cognitive training might yield different levels of performance gains between the DMC and the DNMC task.

The second goal of the present study was to explore the neural mechanisms underlying possible training effects on WM using electrophysiological measures. The event-related potential (ERP) technique has been demonstrated as an effective approach in uncovering the neural processes underlying training-related performance gains. For example, previous research has shown that the changes in N2 and P3 components which might reflect response selection and the allocation of cognitive resources in a task switching or n-back task might underlie performance gains due to multi-domain cognitive training^32–35^. To this end, scalp electroencephalography (EEG) data were recorded from participants while performing the DMC/DNMC task. In the present study, we focused on ERP components (i.e., P2, N2 and P3) which have been widely shown to be related to the WM retrieval phase. Specifically, P2 is a positive component usually appearing around 200 ms after stimulus presentation over the frontal-central scalp region, and past work has suggested that P2 might reflect target detection and attentional allocation in WM^36–38^. The negative-going N2 usually appears around 300 ms post-stimulus and has been proposed to reflect the discrimination of mismatches between the current stimulus and the representation of a stimulus held in memory^38,39^. The final component of interest, P3, which typically occurs around 400–500 ms, indexes many cognitive processes such as attention and WM updating^40–42^. Since previous functional MRI research has shown that training-related changes in WM networks were potentially related to WM updating^12^, we expected to observe training-related changes in the component related to WM updating, i.e., P3.

## Methods

### Participants

Forty-nine healthy older adults were recruited from a local community located in Putuo District, Shanghai. Written informed consent was obtained from each subject before the participation. The study protocol was approved by the Institutional Review Board of Tongji Hospital of Tongji University, and complied with the Declaration of Helsinki. The inclusion criteria included: aged between 70 and 79 years; had at least one year school education; had normal functional capacity and independent living ability in the community; without abnormality in hearing, vision or communication; no major physical disease or psychotic disorders. The participants with obvious cognitive decline, major medical or neurological disorders, or psychiatric disorders were excluded from participation. These participants were different from those in our previous manuscript^23^. They were assigned into two groups, i.e., a multi-domain training group (N = 27) and a control group (N = 22), with matched age, gender and education. Among these participants, 23 (out of 27, 2 lost to follow-up due to illness, 2 refused to do post-training test) in the training group and 16 (out of 22, 4 lost to follow-up due to illness, 2 refused to do post-training test) in the control group completed cognitive assessments and EEG recording during a WM task (see below) at a pre-training (baseline) session and a post-training (follow-up) session (conducted within one month after the end of training). The demographic information of these 39 participants who were included in the final analysis can be found in Table 1. To examine possible impacts caused by the sample size difference between the training (*N* = 23) and control (*N* = 16) group, we conducted additional statistical analyses by randomly selecting 16 participants in the training group. Results were overall similar to that observed using 23 participants in the training group. Therefore, in the following we only reported the results based on 23 participants in the training group.

**Table 1:** Group demographics (mean ± SD).

	Training group (N = 23)	Control group (N = 16)	*p* value
Age	72.8 ± 1.9 (range 70–78)	73.2 ± 1.2 (range 71–75)	0.503^a^
Male/female	15/8	10/6	0.862^b^
Education (years)	9.4 ± 3.5	9.4 ± 2.7	0.954^a^
**MMSE**			
Baseline	27.0 ± 2.8	27.9 ± 1.8	0.242^a^
Follow-up	27.3 ± 1.6^c^	27.8 ± 1.6	0.368^a^
**ADL**			
Baseline	14.1 ± 0.3	14.1 ± 0.3	0.785^a^
Follow-up	14.0 ± 0.2^c^	14.0 ± 0.0	0.401^a^

^a^Independent-samples *t*-test.

^b^Chi-square test.

^c^The data from one participant was not available due to incompliance with the assessment.

### Cognitive interventions and neuropsychological tests

The multi-domain training group received cognitive training at a frequency of twice a week over a period of 12 weeks, resulting in a total of 24 training sessions. Cognitive intervention was provided by two psychiatrists in a face-to-face manner to the 27 individuals in the training group. The percentage of participation in the training sessions dropped from 96% (26/27) at the first session to 63% (17/27) at the twenty-second session, and the mean percentage of participation over the 24 sessions was 76%. The cognitive training program covered the domains of memory (e.g., memorizing pictures and words, adapted from the word learning test and digit span test), reasoning (e.g., the identification of patterns in a group of words, numbers, or pictures, adapted from the Raven's Progressive Matrices), problem-solving strategies (e.g., forming strategies for different tasks) and behavioral exercises (e.g., handwriting and handcrafts). Each session covered one domain, with domains of training counterbalanced across sessions. The training difficulty was fixed within each training session, and participants were required to provide feedback about the difficulty level, perceived usefulness, and interestingness of the session after each session. Such information was subsequently used to adjust the following sessions. Each training session lasted for 60 min. A lecture was presented on the common diseases in older adults during the first 15 min of each session. Participants were then trained for 30 min, with the last 15 min used to consolidate the newly practiced skills. Between every two training sessions, participants in the training group were encouraged to do physical exercise and finish the homework assigned during the previous session (i.e., reading, calligraphy, painting). See Cheng et al.^23^ and Feng et al.^43^ for more training details. Participants in the control group did not receive any cognitive training. Both the multi-domain training group and the control group attended lectures about healthy living once every month. Each lecture lasted for approximately 1 h, resulting in a total of 3 h lecture time for all participants.

All participants received neuropsychological tests at the baseline and follow-up session to evaluate the effects of cognitive training on cognition. The tests included the Chinese version of the Mini-Mental State Examination (MMSE) and Activities of Daily Living (ADL).

### Working memory paradigm and stimuli

**Figure 1:**
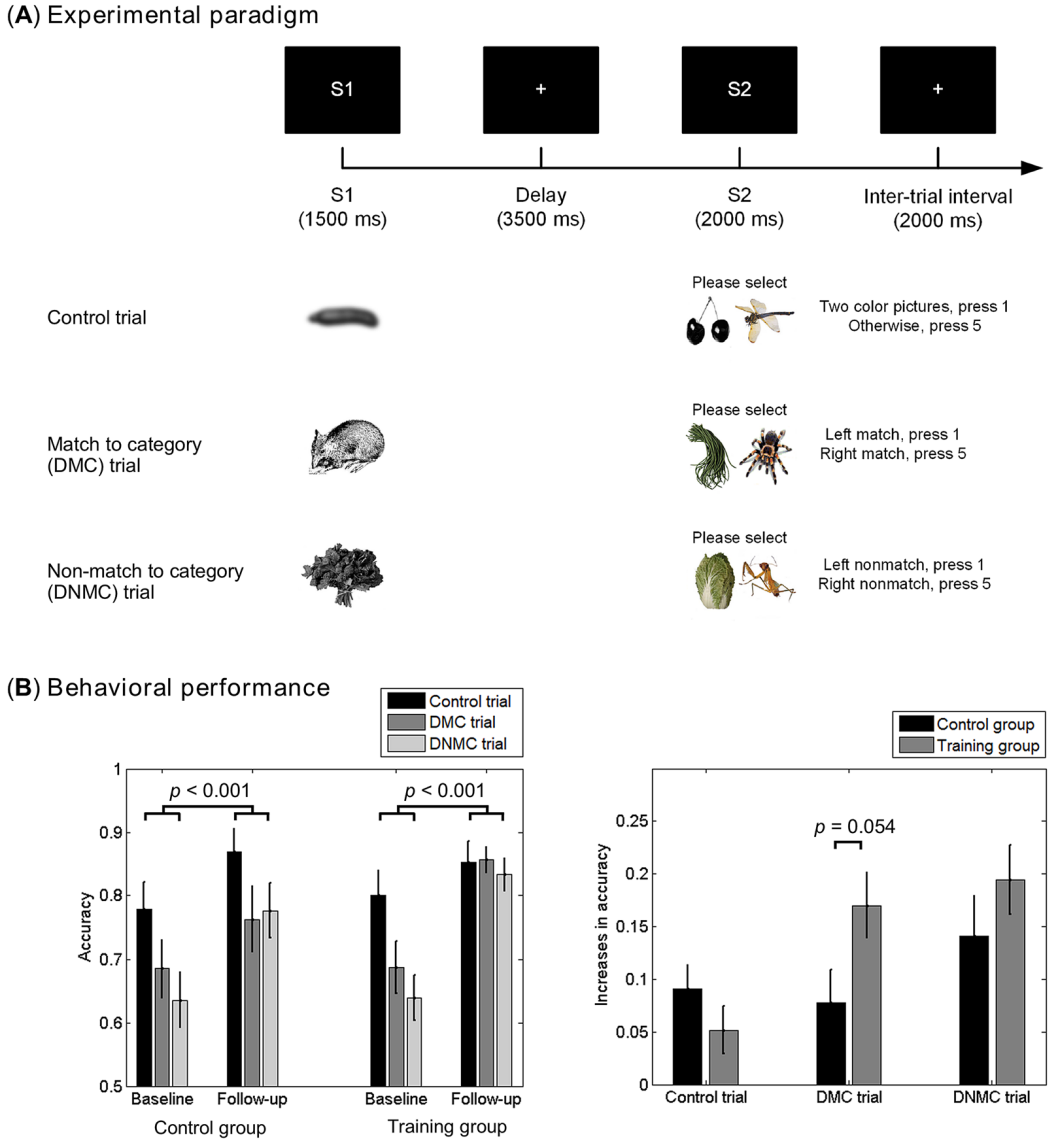
Experimental paradigm (**A**) and behavioral performance (**B**). (**A**) During each trial, a sample stimulus (S1) consisting of an object in black and white was first presented for 1500 ms. After a delay of 3500 ms, a probe stimulus (S2) consisting of two objects either in color or in black and white was presented for 2000 ms. In the control trial, participants must indicate whether the two objects of S2 were both in color or not. In the delayed match or non-match to category (DMC/DNMC) trial, participants must indicate the category of object in which visual hemifield matched (or did not match) that of the sample stimulus. Trials were separated by an inter-trial interval of 2000 ms. (**B**) Participants in both groups showed significant increases in accuracy at the follow-up session relative to the baseline session. Participants in the training group tended to show greater improvements than participants in the control group in the DMC trials. See main text for the details about statistics. Vertical bars indicate mean ± SEM.

Participants performed a visual delayed match or non-match to category (DMC/DNMC) task, a classic experimental paradigm in the study of WM^44^. As Fig. 1A illustrates, each trial began with a sample stimulus (S1) lasting for 1500 ms. After a retention period of 3500 ms, a probe stimulus (S2) was presented for 2000 ms. S1 included a single object in black and white. S2 included two objects in black and white or in color, with one object presented in the left visual hemifield and the other presented in the right visual hemifield. There were 54 different S1 stimuli, which were paired with 54 different S2 stimuli. When S2 was presented, participants were required to indicate which object (referred to as the target object hereafter) in S2 matched (DMC) or did not match (DNMC) the category of the object in S1 by pressing the button ‘1’ when the target object was in the left visual hemifield, and pressing button ‘5’ when the target object was in the right visual hemifield. The location of the target was matched with the location of response button, as the button ‘1’ and button ‘5’ were placed at the left and the right side in front of the participant, respectively. Such a task design could minimize possible conflicts between stimulus and response. In addition to the DMC and the DNMC trials, another type of trials, i.e., the control trial, was included in the experimental design. Participants had to indicate whether the two objects in S2 were both in color (press button ‘1’) or not (press button ‘5’) in the control trials. The inter-trial interval was 2000 ms.

Each session included 9 control trials, followed by 9 DMC trials, and then 9 DNMC trials. The sequence of control, DMC and DNMC trials was fixed across participants, and the goal of this setting was to minimize possible impacts of task instructions for the DMC/DNMC trials on the control trials. After a short training session to get familiar with the task, each participant finished 6 sessions, with a 2–3 min break between every two successive sessions, resulting a total of 54 trials for each trial type. E-Prime 2.0 toolkit (Psychology Software Tools Inc., Sharpsburg, PA, USA) was used for the implementation of the experimental design and the recording of behavioral responses.

### EEG recoding and preprocessing

Scalp EEG data were continuously recorded with a 64-channel EasyCap connected to two BrainAmp DC amplifiers (Brain Products GmbH, Gilching, Germany). The 60 recording electrodes were placed according to the International 10/20 System, including: Fp1, F7, Fp2, F3, FC3, FT7, T7, F8, F4, Fz, FCz, C3, TP7, FT8, FC4, Cz, CPz, CP3, P3, P7, T8, TP8, C4, P8, CP4, P4, Pz, Oz, O1, O2, Fpz, AF3, AF7, F5, AF8, AF4, F1, FC5, F6, F2, FC1, C5, FC6, FC2, C2, C1, CP1, CP5, P5, PO7, PO8, C6, CP6, P6, CP2, PO4, P2, POz, P1, PO3. The AFz was used as the recording ground and the tip of the nose was used as the recoding reference. To monitor ocular activity, the horizontal and vertical electrooculograms (EOGs) were measured by electrodes placed at the outer canthi (LO1, LO2) and above and below the left eye (SO1, IO1). The digitization rate was 1000 Hz and the online anti-aliasing band pass filtering was 0.016–200 Hz. Electrode impedance was kept below 10 kΩ throughout the recording.

Matlab-based EEGLAB^45^ and ERPLAB^46^ toolboxes were used for the offline preprocessing. Raw continuous EEG signal first went through a two-way Butterworth band-pass (0.1–40 Hz) filter with zero phase shift (roll-off slope: 12 dB/oct), then followed by a Parks-McClellan notch filter to minimize the 50 Hz powerline noise. Independent component analysis based on Informax algorithm was then applied to correct ocular artifacts. After that, continuous EEG data were down-sampled to 250 Hz and then segmented into epochs time-locked to S2 (− 200 to 1000 ms post-S2). We note that for the present study, only the retrieval stage of WM was of interest. Therefore, we only analyzed the ERPs elicited by the probe stimulus (S2). Epochs meeting one or more of the following criteria were marked as artifacts and rejected in the following ERP analysis: (1) the maximal voltage difference in any EEG channel exceeded 150 μV within any of the moving windows (width: 200 ms; step: 50 ms) throughout the epoch, examined by a peak-to-peak (maximum *minus* minimum) function, (2) the absolute value of voltage at any time point throughout the whole epoch in any EEG channel exceeded 150 μV, examined by a simple voltage threshold function. After preprocessing, the percentages of rejected epochs with artifacts across participants (mean ± SD) were 1.54 ± 2.96% (baseline session) and 1.37 ± 2.18% (follow-up session) for the training group, and 4.22 ± 8.33% (baseline session) and 1.12 ± 1.50% (follow-up session) for the control group. Only epochs with behaviorally correct responses and without artifacts were included in the following ERP analysis.

### ERP analysis

ERPs elicited by S2 were obtained by averaging all epochs of the same trial type for each session and participant, with the pre-stimulus 200 ms as baseline. According to the literature, P2 and N2 components were typically observed over the frontal-central region, and P3 component was typically observed over the parietal region. We thus defined two regions-of-interest (ROIs), i.e., a frontal-central ROI (Fz, FC1, FCz, FC2, Cz) and a parietal ROI (CPz, P1, Pz, P2, POz). ERPs were then averaged across the electrodes within each ROI. Based on previous studies^38,47,48^ as well as the ERPs observed in the present study, the amplitudes of P2, N2 and P3 components were obtained by averaging the voltages within the windows of 200–250 ms, 300–350 ms and 450–550 ms, respectively. The individual peak latency for each ERP component was identified as the time point of the most negative (N2) or most positive (P2, P3) voltage during the same windows as that used for measuring the mean amplitude. In order to get a reliable measurement of the individual peak latency, individual ERPs were filtered into 0–15 Hz to minimize the interference of high-frequency noise.

### Statistical analysis

Behavioral and ERP data were analyzed using three-way mixed Analysis of Variance (ANOVA) with Session (baseline, follow-up) and Task (control, DMC, DNMC) as within-subject factors, and Group (control, training) as a between-subject factor. We note that because part of the reaction time data were lost due to hardware issues, we only reported accuracy for behavioral performance in the present study. For ERP amplitude data, statistical analysis was performed with the following two steps. First, to examine whether these components were significantly modulated by the WM task and get an overall impression of training-related effects, we conducted an initial 3-way ANOVA with Session (baseline, follow-up) and Task (control, DMC, DNMC) as within-subject factors, and Group (control, training) as a between-subject factor. Second, we calculated the amplitude differences between the DMC/DNMC and the control trials as the task modulation (DMC/DNMC trials *minus* control trials) for each component of interest, participant and session^41^. This could help to isolate the ERP effects related to WM retrieval by minimizing ERP effects related to other processes, e.g., behavioral responses, and also possible impacts of the baseline differences in ERP amplitudes between the two subject groups. A 3-way ANOVA was conducted on such task modulations of ERP amplitudes with Session (baseline, follow-up) and Task (DMC, DNMC) as within-subject factors, and Group (control, training) as a between-subject factor.

For ANOVA, Greenhouse–Geisser adjustment was applied to correct the degrees of freedom when the assumption of sphericity was violated, and the uncorrected degrees of freedom and corrected *p* values were reported in this case. Post hoc tests were conducted using the least significant difference (LSD) method to analyze the significant main effects shown by the ANOVA when appropriate. Independent-samples and paired-samples *t*-tests (2-tailed) were conducted to inform the interactions when necessary. Statistical analysis was performed in SPSS 22.0. Statistical test was considered as significant when *p* < 0.05. All results were presented as mean ± SEM (standard error of the mean) unless otherwise specified.

## Results

### Behavioral results

Mean accuracy was analyzed for each trial type (control, DMC and DNMC), session (baseline, follow-up) and participant (Fig. 1B). We conducted a 3-way ANOVA on accuracy, with Session (baseline, follow-up) and Task (control, DMC and DNMC) as within-subject factors, and Group (control, training) as a between-subject factor. This ANOVA revealed the main effects of Session (*F*_(1,37)_ = 64.787, *p* < 0.001, *η*^2^_p_ = 0.636) and Task (*F*_(2,74)_ = 10.801, *p* < 0.001, *η*^2^_p_ = 0.226), indicating significant task-related modulation and training-related gains in accuracy. A post hoc LSD test showed that the accuracy in the control trials was significantly higher than that in the DMC (*p* = 0.004) and DNMC (*p* < 0.001) trials, and that the accuracy in the DMC trials tended to be higher than that in the DNMC trials (*p* = 0.09). In addition, we observed a significant 2-way interaction of Session × Task (*F*_(2,74)_ = 5.705, *p* = 0.005, *η*^2^_p_ = 0.134) and a marginally significant 3-way interaction of Session × Task × Group (*F*_(2,74)_ = 2.758, *p* = 0.070, *η*^2^_p_ = 0.069), while the interaction of Session × Group (*F*_(1,37)_ = 1.399, *p* = 0.245, *η*^2^_p_ = 0.036) was not significant. Such results indicated that the accuracy gains depended on the trial type and subject group. The main effect of Group was not observed (*F*_(1,37)_ = 0.395, *p* = 0.533, *η*^2^_p_ = 0.011), suggesting that the overall accuracy was not significantly different between the two groups. To further characterize the 3-way interaction, we then performed 2-way ANOVA with Session and Task as within-subject factors in each subject group. We observed the main effect of Session in both the training group (*F*_(1,22)_ = 43.070, *p* < 0.001, *η*^2^_p_ = 0.662) and the control group (*F*_(1,15)_ = 28.627, *p* < 0.001, *η*^2^_p_ = 0.656), and the main effect of Task in both the training group (*F*_(2,44)_ = 3.986, *p* = 0.041, *η*^2^_p_ = 0.153) and the control group (*F*_(2,30)_ = 8.465, *p* = 0.001, *η*^2^_p_ = 0.361). Moreover, a significant interaction of Session × Task was only observed in the training group (*F*_(2,44)_ = 8.695, *p* = 0.001, *η*^2^_p_ = 0.283), which explained the 3-way interaction. Further paired samples *t*-tests suggested that the performance was improved in all three trial types (control trials: *t*_(22)_ =  − 2.215, *p* = 0.037, Cohen’s *d* = 0.462; DMC trials: *t*_(22)_ =  − 5.396, *p* < 0.001, Cohen’s *d* = 1.125, DNMC trials: *t*_(22)_ =  − 5.746, *p* < 0.001, Cohen’s *d* = 1.198), while the magnitude of training-related gains (follow-up *minus* baseline) was larger in the DMC/DNMC trials than that in the control trials (DMC vs. control: *t*_(22)_ = 3.599, *p* = 0.002, Cohen’s *d* = 0.750; DNMC vs. control: *t*_(22)_ = 3.664, *p* = 0.001, Cohen’s *d* = 0.764).

Furthermore, we directly compared the magnitude of training-related performance gains for each trial type between the two subject groups by calculating the differences in accuracy between different sessions (follow-up *minus* baseline). Here we did not correct for multiple comparisons as these were planned comparisons to test pre-defined hypotheses. As Fig. 1B illustrates, participants in the training group showed a trend of greater gains in accuracy than participants in the control group for the DMC and DNMC trials, but not for the control trials. Independent-samples *t*-tests suggested that the differences approached statistical significance for the DMC trials (*t*_(37)_ = 1.990, *p* = 0.054, Cohen’s *d* = 0.648), but not for the DNMC trials (*t*_(37)_ = 1.024, *p* = 0.312, Cohen’s *d* = 0.333) or the control trials (*t*_(37)_ =  − 1.141, *p* = 0.261, Cohen’s *d* = 0.371). Such behavioral results suggest that the 3-month multi-domain cognitive training tended to be more effective in improving WM task performance than only attending health education lectures.

### ERP results

**Figure 2:**
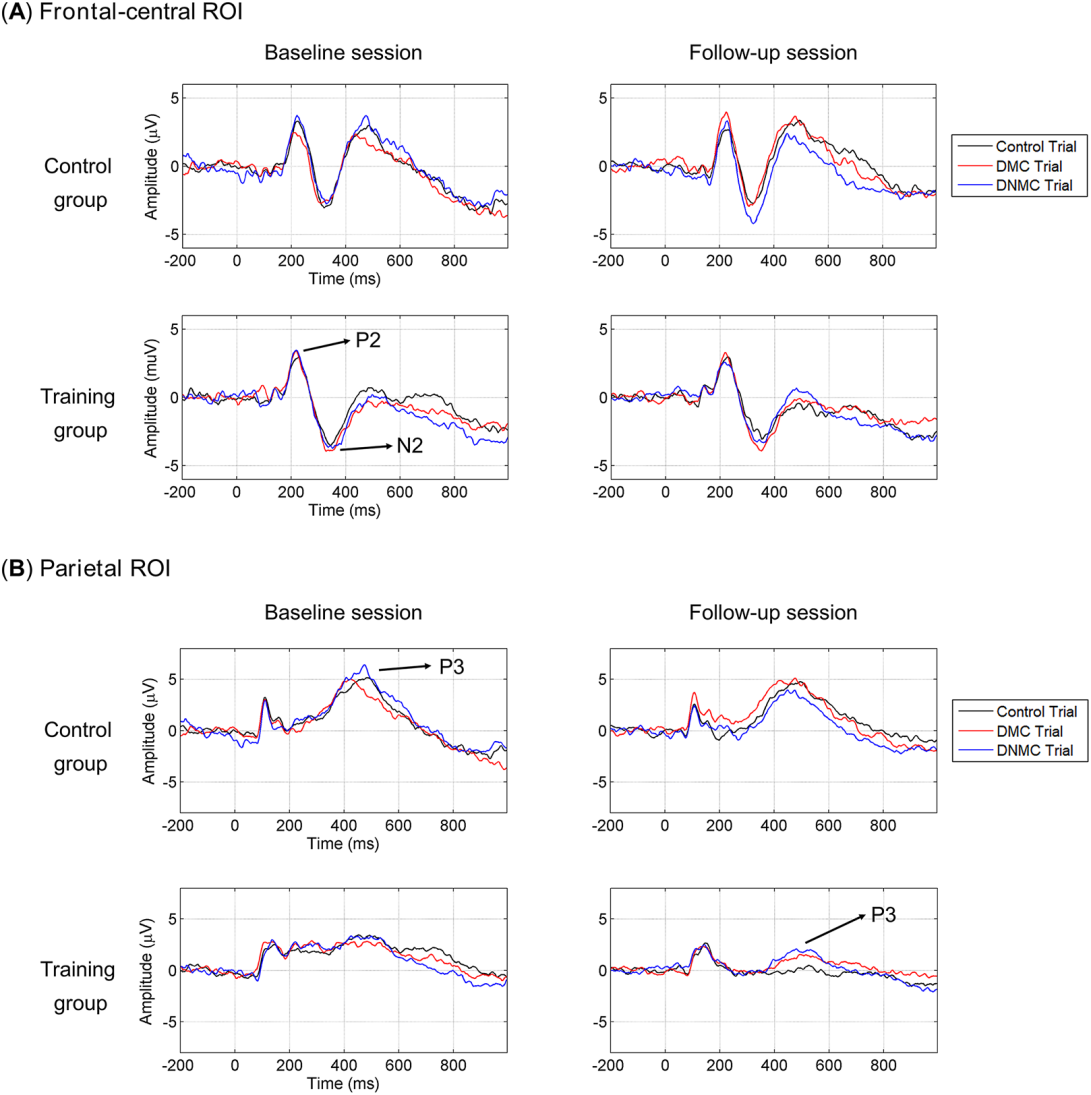
Grand-averaged ERP waveforms time-locked to the probe stimulus (S2) for the control trials, delayed match to category (DMC) trials and delayed non-match to category (DNMC) trials. ERPs were averaged within a frontal-central ROI (**A**) and a parietal ROI (**B**).

**Figure 3:**
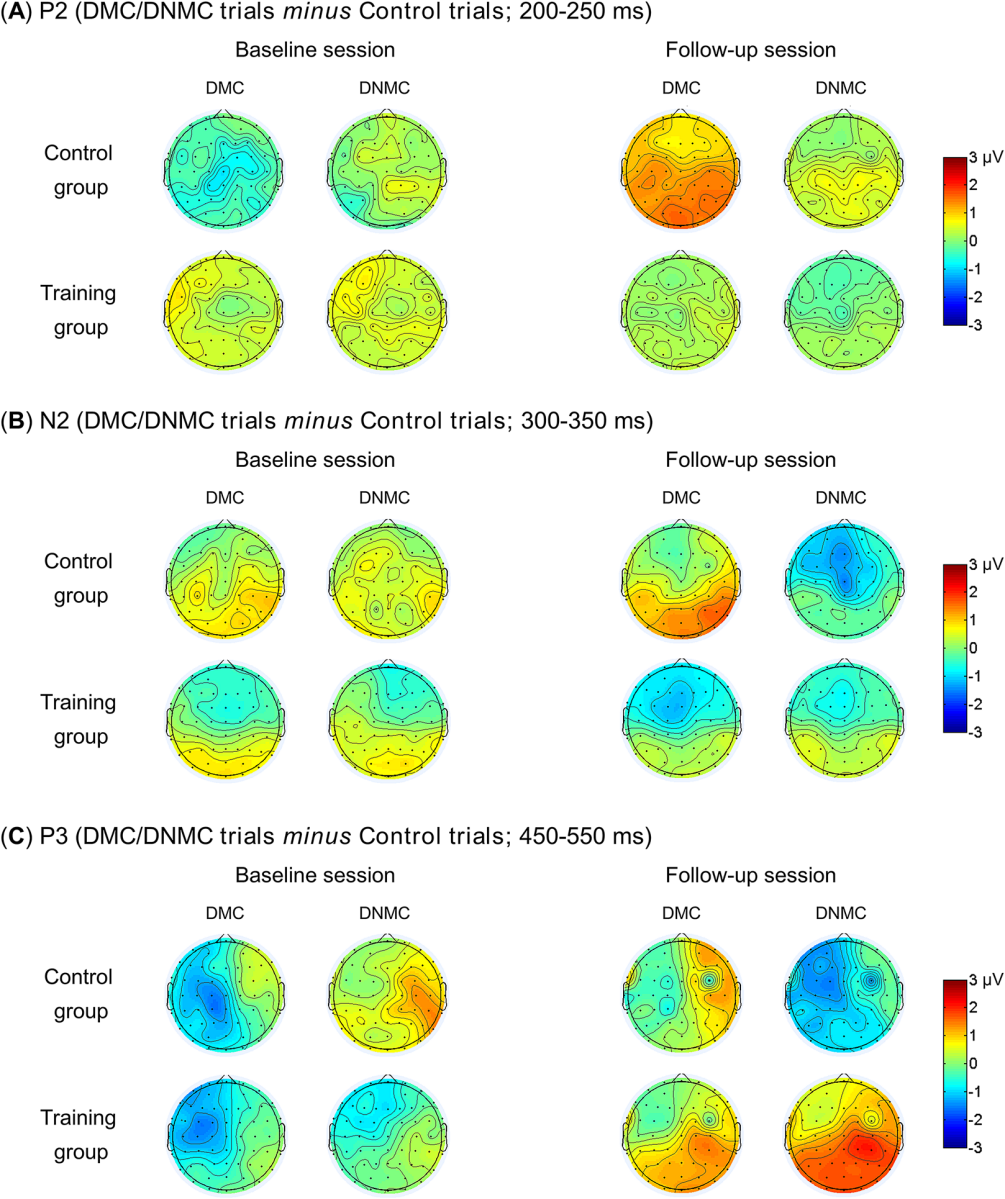
Grand-averaged topographical maps of task modulations (DMC/DNMC trials *minus* control trials) of P2 (**A**), N2 (**B**) and P3 (**C**) averaged within corresponding time windows.

Figure 2 illustrates the grand-averaged ERP waveforms elicited by the probe stimulus (S2) over the frontal-central ROI and the parietal ROI in different sessions and subject groups. Figure 3 illustrates the grand-averaged topographical maps for the task modulations of P2, N2 and P3 component.

#### P2 component

**Figure 4:**
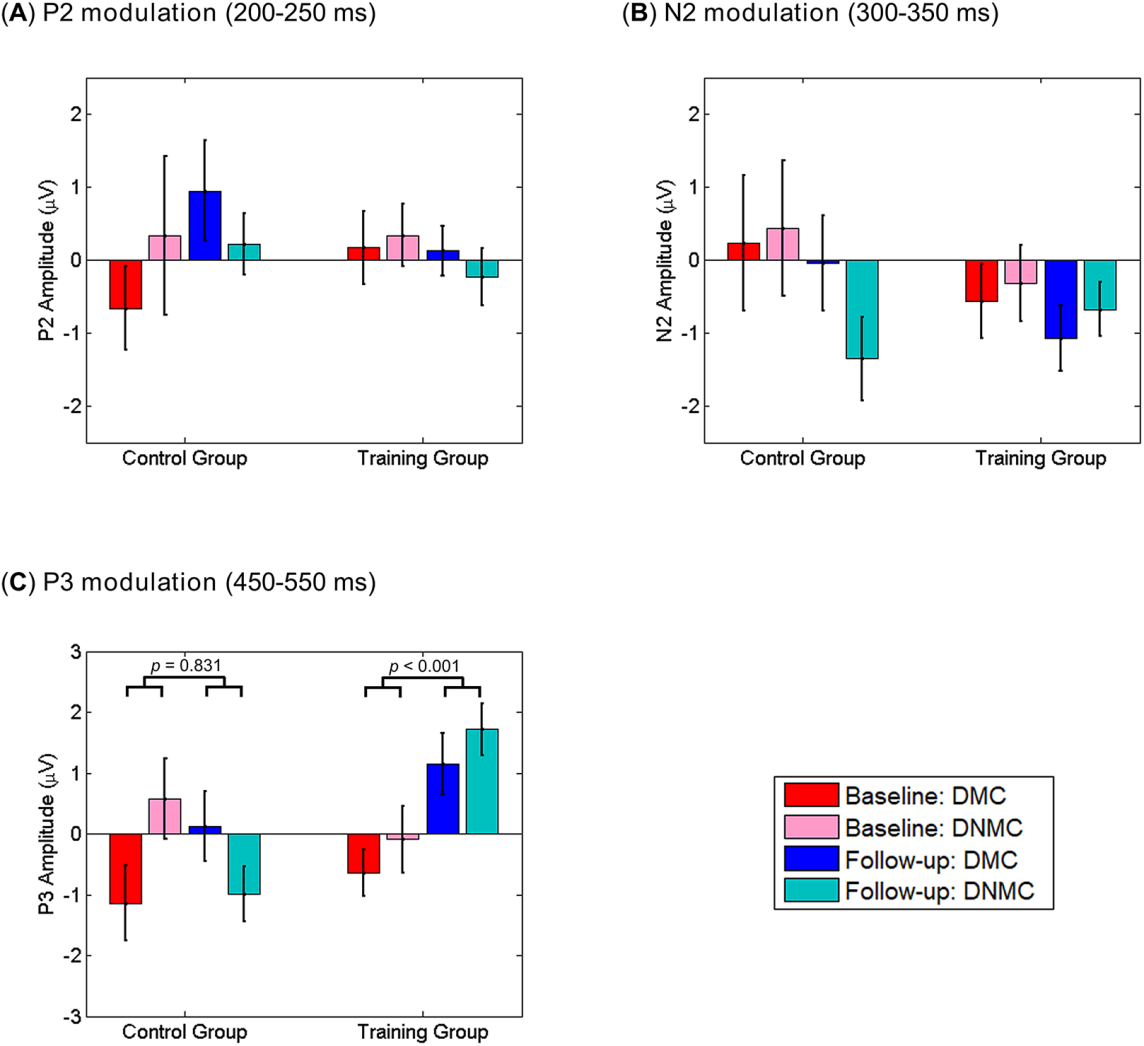
The task modulations (DMC/DNMC trials *minus* control trials) of P2 (**A**), N2 (**B**) and P3 (**C**) amplitudes before and after training in each subject group. Participants in the training group showed significantly (*p* < 0.001) greater task modulation of P3 amplitude after receiving cognitive training (**C**). See main text for the details about statistics. Vertical bars indicate mean ± SEM.

The initial 3-way ANOVA revealed no main effects or significant interactions (all *p*s > 0.1). For the task modulation of P2 amplitude, as illustrated in Fig. 4A, the 3-way ANOVA revealed a marginally significant 2-way interaction of Session × Task (*F*_(1,37)_ = 3.615, *p* = 0.065, *η*^2^_p_ = 0.089). We thus performed additional 2-way ANOVA with Session as a within-subject factor and Group as a between-subject factor for the DMC and the DNMC trials separately. However, no main effects or significant interactions were observed for either type of trials (all *p*s > 0.1). Such results suggest that P2 was not significantly modulated by the WM task or cognitive training.

For the peak latency of P2, the 3-way ANOVA revealed only a significant interaction of Session × Group (*F*_(1,37)_ = 4.109, *p* = 0.050, *η*^2^_p_ = 0.100), without other main effects or significant interactions (all *p*s > 0.4). To inform this interaction, we then performed additional 2-way ANOVA with Session and Task as within-subject factors in each subject group. For the training group, the main effect of Session (*F*_(1,22)_ = 3.259, *p* = 0.085, *η*^2^_p_ = 0.129) approached statistical significance, suggesting a trend of longer P2 latency in the follow-up session compared with the baseline session. This trend level main effect of Session was not observed for the control group (*F*_(1,15)_ = 1.290, *p* = 0.274, *η*^2^_p_ = 0.079). In addition, no other main effects or significant interactions were observed in either group (all *p*s > 0.6).

#### N2 component

The initial 3-way ANOVA revealed no main effects or significant interactions (all *p*s > 0.1). For the task modulation of N2 amplitude, as illustrated in Fig. 4B, the 3-way ANOVA revealed a marginally significant 2-way interaction of Task × Group (*F*_(1,37)_ = 3.101, *p* = 0.086, *η*^2^_p_ = 0.077). We then performed additional 2-way ANOVA with Session and Task as within-subject factors in each subject group. However, no main effects or significant interactions were observed in either subject group (all *p*s > 0.1). Such results suggest that N2 was not significantly modulated by the WM task or cognitive training.

For the peak latency of N2, the 3-way ANOVA revealed a main effect of Group (*F*_(1,37)_ = 8.246, *p* = 0.007, *η*^2^_p_ = 0.182), suggesting that the training group had significantly longer N2 latency than the control group. No other main effects or significant interactions were observed (all *p*s > 0.2).

#### P3 component

The initial 3-way ANOVA revealed a main effect of Session (*F*_(1,37)_ = 4.387, *p* = 0.043, *η*^2^_p_ = 0.106), indicating that P3 amplitudes were smaller in the follow-up session compared with the baseline session. The main effect of Group (*F*_(1,37)_ = 3.268, *p* = 0.079, *η*^2^_p_ = 0.081) approached statistical significance, indicating that participants in the control group tended to show larger P3 amplitudes than participants in the training group. Besides, we observed a significant 2-way interaction of Session × Task (*F*_(2,74)_ = 4.381, *p* = 0.024, *η*^2^_p_ = 0.106) and a significant 3-way interaction of Session × Task × Group (*F*_(2,74)_ = 4.982, *p* = 0.009, *η*^2^_p_ = 0.119), while the 2-way interaction of Session × Group (*F*_(1,37)_ = 2.094, *p* = 0.156, *η*^2^_p_ = 0.054) was not significant. To inform such interactions and test whether P3 amplitude was modulated by the WM task, we performed one-way ANOVA with Task as the within-subject factor for each subject group and session separately. For participants in the training group, the main effect of Task was significant at the follow-up session (*F*_(2,44)_ = 6.681, *p* = 0.003, *η*^2^_p_ = 0.233) while was not significant at the baseline session (*p* > 0.4). A post hoc LSD test suggested that at the follow-up session, the DMC and DNMC trials elicited larger P3 than the control trials (*p* = 0.036 and *p* = 0.001). For participants in the control group, the main effect of Task was not significant at the baseline session (*p* > 0.09) while approached significance at the follow-up session (*F*_(2,30)_ = 2.792, *p* = 0.077, *η*^2^_p_ = 0.157). A post hoc LSD test suggested that at the follow-up session, the DNMC trials elicited smaller P3 than the control and DMC trials (*p* = 0.053 and *p* = 0.031). Overall, such results indicate that although P3 was not significantly modulated by the WM task for participants in both groups at the baseline session, the task modulation of P3 (i.e., larger amplitudes in the DMC/DNMC trials than in the control trials) was regained after cognitive training for participants in the training group.

For the task modulation of P3 amplitude, as illustrated in Fig. 4C, the 3-way ANOVA revealed a main effect of Session (*F*_(1,37)_ = 5.832, *p* = 0.021, *η*^2^_p_ = 0.136) and a marginal main effect of Group (*F*_(1,37)_ = 3.233, *p* = 0.080, *η*^2^_p_ = 0.080), suggesting significantly greater P3 modulation in the follow-up session compared with the baseline session, and a trend toward greater P3 modulation for the training group compared with the control group. Moreover, we observed a significant 2-way interaction of Session × Group (*F*_(1,37)_ = 8.122, *p* = 0.007, *η*^2^_p_ = 0.180), a marginally significant 2-way interaction of Session × Task (*F*_(1,37)_ = 3.927, *p* = 0.055, *η*^2^_p_ = 0.096), and a marginally significant 3-way interaction of Session × Task × Group (*F*_(1,37)_ = 3.998, *p* = 0.053, *η*^2^_p_ = 0.098). To characterize these interactions, we performed additional 2-way ANOVA with Session and Task as within-subject factors in each subject group. For the training group, the main effect of Session (*F*_(1,22)_ = 32.085, *p* < 0.001, *η*^2^_p_ = 0.593) was observed, indicating that participants showed significantly greater P3 modulation after cognitive training. No other main effects or significant interactions were observed in the training group (all *p*s > 0.1). In contrast, for the control group, the main effect of Session was not observed (*F*_(1,15)_ = 0.047, *p* = 0.831, *η*^2^_p_ = 0.003), while there was a significant interaction of Session × Task (*F*_(1,15)_ = 6.581, *p* = 0.022, *η*^2^_p_ = 0.305). Further *t*-tests indicated that participants in the control group showed marginally smaller P3 modulation in the follow-up session relative to the baseline session for the DNMC trials (*t*_(15)_ = 1.857, *p* = 0.083, Cohen’s *d* = 0.464), while the direction of this difference was reversed, although not significant, for the DMC trials (*t*_(15)_ =  − 1.374, *p* = 0.190, Cohen’s *d* = 0.343). Overall, these results suggest that participants in the training group showed enhanced task modulation of P3 amplitudes after receiving cognitive training, while such effects were not observed for participants in the control group.

For the peak latency of P3, the 3-way ANOVA revealed a main effect of Group (*F*_(1,37)_ = 5.082, *p* = 0.030, *η*^2^_p_ = 0.121), suggesting that the training group had significantly longer P3 latency than the control group. No other main effects or significant interactions were observed (all *p*s > 0.09).

## Discussion

In the present study, we examined whether a previously established multi-domain cognitive training program that was not designed to specifically target WM could improve behavioral performance and ERP responses of WM in healthy older adults. We assigned healthy older participants from a local community into a training group who completed 3-month multi-domain cognitive training and a control group who only attended health education lectures during the same period. In both pre-training (baseline) and post-training (follow-up) sessions, behavioral and EEG data were recorded from participants while performing an untrained WM task. By comparing the performance between the baseline session and the follow-up session, we found that participants in the training group tended to show greater gains in behavioral accuracy than participants in the control group. For the ERP data, we observed a significantly enhanced task-related modulation of P3 amplitude after cognitive training in the training group, but not in the control group. Moreover, no significant training-related effects were observed for other ERP components, i.e., P2 and N2. Altogether, these results suggest that the multi-domain cognitive training program that was not designed to specifically target WM is a promising approach to improve WM performance in healthy older adults, and that the enhancement of P3 modulation which was associated with WM updating might reflect the underlying neural substrate.

The behavioral data in the present study confirmed our hypothesis that the multi-domain cognitive training program can improve WM performance in healthy older adults. An interesting finding is that participants in both groups showed significant improvements in the follow-up session relative to the baseline session. It might indicate a practice effect induced by repeated testing in addition to the performance gains related to cognitive training^33^. However, by directly comparing the net increase of accuracy between the two groups (Fig. 1B), we found that participants in the training group showed greater improvements than participants in the control group, although this group difference was only marginally significant for the DMC trials (*p* = 0.054). In contrast, for the DNMC trials, despite a trend of greater improvements in the training group relative to the control group, there was no significant differences between the two groups (*p* = 0.312). Such results might be due to the relatively small sample size (see discussion below), while it might also reflect the differences between the DMC and DNMC trials. Specifically, DNMC is harder than DMC for adult humans^29^, which was supported by our results that participants showed a trend of lower accuracy in DNMC trials than in DMC trials (*p* = 0.09). Moreover, as shown in Fig. 1B, our findings suggested that for participants in the training group, the performance in the DMC and DNMC trials was promoted to similar levels compared with the control trials at the follow-up session, which might indicate that the performance in DMC/DNMC trials might reach the ceiling of improvement. This might partly explain why the performance gains in DMC trials, not in DNMC trials, only approached statistical significance when comparing between the two subject groups.

Our ERP results suggest that the task modulation of P3 amplitude was enhanced for participants who received 3-month multi-domain cognitive training. Such enhanced P3 modulation, however, was not observed for participants in the control group who only attended lectures about healthy living. Combined with the behavioral finding, our results suggest that P3 modulation might reflect the neural substrate of training effects on WM performance in older adults. Although the functional significance of P3 is still not precisely known, many studies have suggested that P3 observed in WM tasks might reflect the updating of WM^40,42,49^. At the same time, P3 has been also shown to be sensitive to normal aging^50^, and age-related reductions in P3 amplitude have been reported by many WM studies^51–53^. Consistent with the literature, we found that P3 amplitude was not significantly modulated by the WM task (i.e., the main effect of Task was not observed) at the baseline session for participants in either group. At the follow-up session, participants in the training group showed significant P3 modulation (i.e., larger P3 amplitudes in the DMC/DNMC trials than in the control trials), while such modulation was still absent for participants in the control group. This pattern of results explained why participants in the training group showed significantly greater task modulation of P3 than participants in the control group (*p* < 0.001; Fig. 4C).

We did not observe significant training effects on the shorter-latency components, i.e., P2 or N2. This finding might indicate that the neural processes underlying P2 and N2 were relatively difficult to change through the multi-domain cognitive training applied in the present study^23^. However, it did not imply that the earlier neural processes could not be altered by specifically designed cognitive training. For example, Anne and colleagues have shown that visual discrimination training over a three to five week period could modify early visual processing during stimulus encoding, which could further predict the improvement in WM accuracy^54^. Nevertheless, the present finding still contrasts with previous studies which showed that multi-domain cognitive training could increase the N2 amplitude in a task switching paradigm^32–34^. This disparity might be explained by different cognitive processes that could elicit the N2 effect. Specifically, it has been proposed that the N2 should reflect the control-related or mismatch-related processes^39^. The control-related processes are most involved in cognitive tasks with fast sequence of stimuli, high conflict (e.g., the Stroop task) and time pressure, and the enhanced N2 has been interpreted as the neural correlate of the improved performance after receiving cognitive training^32,34^. In contrast, for the present study, no fast stimulus sequence or response interference was induced in the DMC/DNMC trials, and thus the N2 should only reflect the discrimination of mismatches between the current stimulus and a mental template stored in WM^38,39,55^. When combined with previous research, our findings might indicate that the control-related processes are more likely to be modified through multi-domain cognitive training compared with mismatch-related processes that are required by WM retrieval.

The cognitive intervention applied in the present study covered the domains of memory, reasoning, problem-solving strategies and behavioral exercises. The memory-related training materials and procedures were adopted from neuropsychological tests, e.g., the word learning test and digit span test. It has been suggested that such tests mainly involved short-term memory (STM), which was regarded as a subcomponent of the larger WM system^28^. Specifically, STM refers to the simple temporary storage of information, while WM implies a combination of storage and manipulation^6^. Thus, it might not be appropriate to consider the training effects in the present study as near transfer. However, considering that WM plays a fundamental role in general cognition, the training materials and procedures included in the present study should inevitably cover, at least to some extent, WM and its interaction with other cognitive processes. In this case, it might be difficult to make a quantitative distinction between near and far transfer. Nevertheless, the current results indicate that the improvement of WM and related neural processing after receiving multi-domain cognitive training might underlie the improvement of a variety of cognitive functions that has been demonstrated in our previous work^23^.

The following limitations need to be considered when interpreting the current findings. Firstly, the sample size in the present study was relatively small, and cognitive assessors were not blinded to the intervention assignment, which may reduce statistical power and prevent the generalization of results to the general population. The sample size estimation was based on the consideration that ~ 20 participants for each group was commonly seen in previous WM studies that compared ERPs between different participant groups^48,56,57^. However, the relatively high drop-out rate warrants larger sample sizes in future studies. Furthermore, considering that the effects of the multi-domain cognitive training have been demonstrated in our previous study using the randomized controlled design^23^, the intervention assignment of participants into the two groups was not randomized in the present study, but was in order to match their age, gender and education levels. Nevertheless, larger sample sizes would also help to uncover the baseline differences in more detail. Here we observed significant or marginally significant baseline differences in ERPs (i.e., N2 latency, P3 amplitude and latency) between the training and control participants, although we tried to minimize possible impacts of such baseline differences by calculating the amplitude differences between the DMC/DNMC and the control trials. It can be speculated that the baseline differences in ERPs might be prevented by the random allocation of training and control participants instead of the matching procedure applied in the present study. Moreover, larger sample sizes might also enable the investigation of inter-individual differences in training-related performance gains and associated neural changes^58^, which would allow the development of more effective and targeted training protocols in the future. Lastly, the reaction time data were lost due to hardware issues, which restricted the understanding of possible training effects on behavioral measures, e.g., lower RT variability after cognitive training^32^. In spite of the above limitations, the present study, for the first time, demonstrated the positive effect of multi-domain cognitive training on WM performance and possible neural mechanisms using ERPs based on a Chinese community-living older participant sample. In sum, future studies with the randomized controlled design, double-blind assessments and relatively larger sample sizes are required to replicate and further extend the current findings.

In conclusion, the present study extends our understanding of the behavioral effects and neural responses of WM associated with a multi-domain cognitive training program that was not designed to specifically target WM. We showed that this 3-month multi-domain cognitive training had beneficial effects on the WM performance in healthy older adults, and that these training-related performance gains were likely mediated by an enhanced modulation of P3 which was believed to reflect WM updating.

## Data Availability

The datasets that support the findings of the present study are available from the corresponding author upon reasonable request.

## References

[1] Salthouse T (2012). Consequences of age-related cognitive declines. Ann. Rev. Psychol..

[2] Park DC, Reuter-Lorenz P (2009). The adaptive brain: aging and neurocognitive scaffolding. Annu. Rev. Psychol..

[3] Gutchess A (2014). Plasticity of the aging brain: new directions in cognitive neuroscience. Science.

[4] Ballesteros S, Kraft E, Santana S, Tziraki C (2015). Maintaining older brain functionality: a targeted review. Neurosci. Biobehav. Rev..

[5] Anguera JA (2013). Video game training enhances cognitive control in older adults. Nature.

[6] Baddeley A (2012). Working memory: theories, models, and controversies. Annu. Rev. Psychol..

[7] Klingberg T (2010). Training and plasticity of working memory. Trends in cognitive sciences.

[8] Iordan AD (2020). Neural correlates of working memory training: Evidence for plasticity in older adults. NeuroImage.

[9] Stepankova H (2014). The malleability of working memory and visuospatial skills: a randomized controlled study in older adults. Dev. Psychol..

[10] Dahlin E, Nyberg L, Backman L, Neely AS (2008). Plasticity of executive functioning in young and older adults: immediate training gains, transfer, and long-term maintenance. Psychol. Aging.

[11] McAvinue LP (2013). An evaluation of a working memory training scheme in older adults. Front. Aging Neurosci..

[12] Heinzel S (2016). Neural correlates of training and transfer effects in working memory in older adults. NeuroImage.

[13] Buschkuehl M (2008). Impact of working memory training on memory performance in old-old adults. Psychol. Aging.

[14] Richmond LL, Morrison AB, Chein JM, Olson IR (2011). Working memory training and transfer in older adults. Psychol. Aging.

[15] Teixeira-Santos AC (2019). Reviewing working memory training gains in healthy older adults: a meta-analytic review of transfer for cognitive outcomes. Neurosci. Biobehav. Rev..

[16] Barnett SM, Ceci SJ (2002). When and where do we apply what we learn? A taxonomy for far transfer. Psychol. Bull..

[17] ShawnGreen C (2019). Improving methodological standards in behavioral interventions for cognitive enhancement. J. Cognit. Enhanc. Towards Integr. Theory Pract..

[18] Strobach T, Huestegge L (2017). Evaluating the effectiveness of commercial brain game training with working-memory tasks. J. Cognit. Enhanc. Towards Integr. Theory Pract..

[19] Karbach J, Verhaeghen P (2014). Making working memory work: a meta-analysis of executive-control and working memory training in older adults. Psychol. Sci..

[20] Melby-Lervag M, Redick TS, Hulme C (2016). Working memory training does not improve performance on measures of intelligence or other measures of "far transfer": evidence from a meta-analytic review. Perspect. Psychol. Sci. J. Assoc. Psychol. Sci..

[21] Neely, A. S. & Nyberg, L. in *Working Memory and Ageing* 95–112 (Psychology Press, 2014).

[22] Eckroth-Bucher M, Siberski J (2009). Preserving cognition through an integrated cognitive stimulation and training program. Am. J. Alzheimer's Dis. Other Dementias.

[23] Cheng Y (2012). The effects of multi-domain versus single-domain cognitive training in non-demented older people: a randomized controlled trial. BMC Med..

[24] Cao W (2016). Effects of cognitive training on resting-state functional connectivity of default mode, salience, and central executive networks. Front. Aging Neurosci..

[25] Luo C (2016). The lateralization of intrinsic networks in the aging brain implicates the effects of cognitive training. Front. Aging Neurosci..

[26] Li T (2016). Cognitive training can reduce the rate of cognitive aging: a neuroimaging cohort study. BMC Geriatrics.

[27] Jiang L (2016). Cortical thickness changes correlate with cognition changes after cognitive training: evidence from a chinese community study. Front. Aging Neurosci..

[28] Shipstead Z, Redick TS, Engle RW (2012). Is working memory training effective?. Psychol. Bull..

[29] Elliott R, Dolan RJ (1999). Differential neural responses during performance of matching and nonmatching to sample tasks at two delay intervals. J. Neurosci. Off. J. Soc. Neurosci..

[30] Gaffan D, Gaffan EA, Harrison S (1984). Effects of fornix transection on spontaneous and trained non-matching by monkeys. Q. J. Exper. Psychol. B Compar. Physiol. Psychol..

[31] Maatoug R (2019). Performance in delayed non-matching to sample task predicts the diagnosis of obsessive-compulsive disorder. Transl. Psychiatry.

[32] Gajewski PD, Falkenstein M (2012). Training-induced improvement of response selection and error detection in aging assessed by task switching: effects of cognitive, physical, and relaxation training. Front. Hum. Neurosci..

[33] Kuper K, Gajewski PD, Frieg C, Falkenstein M (2017). A randomized controlled erp study on the effects of multi-domain cognitive training and task difficulty on task switching performance in older adults. Front. Hum. Neurosci..

[34] Gajewski PD, Freude G, Falkenstein M (2017). Cognitive training sustainably improves executive functioning in middle-aged industry workers assessed by task switching: a randomized controlled ERP study. Front. Hum. Neurosci..

[35] Gajewski PD, Falkenstein M (2018). ERP and behavioral effects of physical and cognitive training on working memory in aging: a randomized controlled study. Neural Plast..

[36] Finnigan S, O'Connell RG, Cummins TD, Broughton M, Robertson IH (2011). ERP measures indicate both attention and working memory encoding decrements in aging. Psychophysiology.

[37] Lijffijt M (2009). P50, N100, and P200 sensory gating: relationships with behavioral inhibition, attention, and working memory. Psychophysiology.

[38] Morrison C, Taler V (2020). ERP measures of the effects of age and bilingualism on working memory performance. Neuropsychologia.

[39] Folstein JR, Van Petten C (2008). Influence of cognitive control and mismatch on the N2 component of the ERP: a review. Psychophysiology.

[40] Polich J (2007). Updating P300: an integrative theory of P3a and P3b. Clin. Neurophysiol. Off. J. Int. Feder. Clin. Neurophysiol..

[41] Luck SJ (2014). An introduction to the event-related potential technique.

[42] Vogel EK, Luck SJ (2002). Delayed working memory consolidation during the attentional blink. Psychon. Bull. Rev..

[43] Feng W, Li C, Chen Y, Cheng Y, Wu W (2014). Five-year follow-up study of multi-domain cognitive training for healthy elderly community members. Shanghai Arch Psychiatry.

[44] Braunlich K, Gomez-Lavin J, Seger CA (2015). Frontoparietal networks involved in categorization and item working memory. NeuroImage.

[45] Delorme A, Makeig S (2004). EEGLAB: an open source toolbox for analysis of single-trial EEG dynamics including independent component analysis. J. Neurosci. Methods.

[46] Lopez-Calderon J, Luck SJ (2014). ERPLAB: an open-source toolbox for the analysis of event-related potentials. Front. Hum. Neurosci..

[47] Getzmann S, Wascher E, Schneider D (2018). The role of inhibition for working memory processes: ERP evidence from a short-term storage task. Psychophysiology.

[48] Pinal D, Zurron M, Diaz F (2015). An event related potentials study of the effects of age, load and maintenance duration on working memory recognition. PLoS ONE.

[49] Verleger R (2020). Effects of relevance and response frequency on P3b amplitudes: review of findings and comparison of hypotheses about the process reflected by P3b. Psychophysiology.

[50] Polich J (1996). Meta-analysis of P300 normative aging studies. Psychophysiology.

[51] McEvoy LK, Pellouchoud E, Smith ME, Gevins A (2001). Neurophysiological signals of working memory in normal aging. Brain Res. Cogn. Brain Res..

[52] Saliasi E, Geerligs L, Lorist MM, Maurits NM (2013). The relationship between P3 amplitude and working memory performance differs in young and older adults. PLoS ONE.

[53] Lubitz AF, Niedeggen M, Feser M (2017). Aging and working memory performance: electrophysiological correlates of high and low performing elderly. Neuropsychologia.

[54] Berry AS (2010). The influence of perceptual training on working memory in older adults. PLoS ONE.

[55] Patel SH, Azzam PN (2005). Characterization of N200 and P300: selected studies of the event-related potential. Int. J. Med. Sci..

[56] Gazzaley A (2008). Age-related top-down suppression deficit in the early stages of cortical visual memory processing. Proc. Natl. Acad. Sci. USA.

[57] Zanto TP, Toy B, Gazzaley A (2010). Delays in neural processing during working memory encoding in normal aging. Neuropsychologia.

[58] Brehmer Y (2011). Neural correlates of training-related working-memory gains in old age. NeuroImage.

