# Author Correction: Effects of multi-domain cognitive training on working memory retrieval in older adults: behavioral and ERP evidence from a Chinese community study

**DOI:** 10.1038/s41598-021-88990-2

**Published:** 2021-04-23

**Authors:** Xiangfei Hong, You Chen, Jijun Wang, Yuan Shen, Qingwei Li, Binglei Zhao, Xiaoli Guo, Wei Feng, Wenyuan Wu, Chunbo Li

**Affiliations:** 1 Shanghai Key Laboratory of Psychotic Disorders, Shanghai Mental Health Center, Shanghai Jiao Tong University School of Medicine, 600 Wan Ping Nan Road, Shanghai, 200030 People’s Republic of China; 2 Shanghai Yangpu District Mental Health Center, Shanghai, 200090 People’s Republic of China; 3 CAS Center for Excellence in Brain Science and Intelligence Technology (CEBSIT), Chinese Academy of Science, Shanghai, People’s Republic of China; 4 Brain Science and Technology Research Center, Shanghai Jiao Tong University, Shanghai, 200030 People’s Republic of China; 5 Institute of Psychology and Behavioral Science, Shanghai Jiao Tong University, Shanghai, 200030 People’s Republic of China; 6 Department of Psychiatry, Shanghai Tenth People’s Hospital, Tongji University School of Medicine, Shanghai, 200072 People’s Republic of China; 7 Department of Psychiatry, Tongji Hospital, Tongji University, Shanghai, 200065 People’s Republic of China; 8 School of Biomedical Engineering, Shanghai Jiao Tong University, Shanghai, People’s Republic of China; 9 Department of Psychological Medicine, Renji Hospital, Shanghai Jiao Tong University School of Medicine, 160 Pujian Road, Shanghai, 200127 People’s Republic of China

Correction to: *Scientific Reports* 10.1038/s41598-020-79784-z, published online 13 January 2021

The original version of this Article contained an error in Affiliation 3, which was incorrectly given as ‘CAS Center for Excellence in Brain Science and Intelligence Technology (CEBSIT), Chinese Academy of Science, Wuhan, People’s Republic of China’. The correct affiliation is listed below:

CAS Center for Excellence in Brain Science and Intelligence Technology (CEBSIT), Chinese Academy of Science, Shanghai, People’s Republic of China.

In addition, in the Methods section under subheading “Participants”,

“The study protocol was approved by the Institutional Review Board of Shanghai Mental Health Center, and complied with the Declaration of Helsinki.”

now reads:

“The study protocol was approved by the Institutional Review Board of Tongji Hospital of Tongji University, and complied with the Declaration of Helsinki.”

This has now been corrected in the HTML and PDF versions of this Article.

